# Ovine Toll-like Receptor 9 (*TLR9*) Gene Variation and Its Association with Flystrike Susceptibility

**DOI:** 10.3390/ani11123549

**Published:** 2021-12-14

**Authors:** Xiu Liu, Huitong Zhou, Hua Gong, Wenting Liu, Qian Fang, Yuzhu Luo, Jiqing Wang, Shaobin Li, Jiang Hu, Jonathan G. H. Hickford

**Affiliations:** 1 Gansu Key Laboratory of Herbivorous Animal Biotechnology, College of Animal Science and Technology, Gansu Agricultural University, Lanzhou 730070, China; liuxiu@gsau.edu.cn (X.L.); wangjq@gsau.edu.cn (J.W.); lisb@gsau.edu.cn (S.L.); huj@gsau.edu.cn (J.H.); 2 Department of Agricultural Sciences, Faculty of Agriculture and Life Sciences, Lincoln University, Lincoln 7647, New Zealand; Huitong.Zhou@lincoln.ac.nz (H.Z.); gonghua3000@gmail.com (H.G.); fangq@lincoln.ac.nz (Q.F.); 3 School of Public Health, Hubei University of Medicine, Shiyan 442000, China; liuwentingnz@outlook.com

**Keywords:** Toll-like receptor 9, flystrike, resistance, susceptible, ovine

## Abstract

**Simple Summary:**

Flystrike is a major ectoparasitic disease of sheep and it creates both an economic impact and welfare issue for the sheep industry. Several factors control the responses of sheep to flystrike, and among these, immune response is regarded as an important factor. Toll-like receptors (TLRs) plays a crucial role in the innate immune system by recognizing pathogen-associated molecular patterns derived from various microbes. Of these receptors, TLR9 recognises unmethylated cytosine-phosphate-guanine (CpG) motifs that are known to be prevalent in bacterial genomes and are also reported in Dipteran insects, including *Lucilia cuprina*, one of the main species associated with flystrike in sheep. In this study, we used a polymerase chain reaction-single strand conformation polymorphism (PCR-SSCP) technique to investigate *TLR9* variation in sheep with and without flystrike, and found that variation in a gene region containing the coding sequence of the putative CpG-DNA binding domain was associated with the likelihood of flystrike occurrence. This suggests that variation in ovine *TLR9* may affect a sheep’s response to flystrike.

**Abstract:**

Toll-like receptors (TLRs) are a family of proteins that play a role in innate immune responses by recognising pathogen-associated molecular patterns derived from various microbes. Of these receptors, TLR9 recognises bacterial and viral DNA containing unmethylated cytosine-phosphate-guanine (CpG) motifs, and variation in *TLR9* has been associated with resistance to various infectious diseases. Flystrike is a problem affecting the sheep industry globally and the immune response of the sheep has been suggested as one factor that influences the response to the disease. In this study, variation in ovine *TLR9* from 178 sheep with flystrike and 134 sheep without flystrike was investigated using a polymerase chain reaction-single strand conformation polymorphism (PCR-SSCP) approach. These sheep were collected from both commercial and stud farms throughout New Zealand and they were of 13 different breeds, cross-breds and composites. Four alleles of *TLR9* were detected, including three previously identified alleles (*01, *02 and *03) and a new allele (*04). In total six single nucleotide polymorphisms (SNPs) were found. Of the three common alleles in the sheep studied, the presence of *03 was found to be associated with a reduced likelihood of flystrike being present (OR = 0.499, *p* = 0.024). This suggests that variation in ovine *TLR9* may affect a sheep’s response to flystrike, and thus the gene may have value as a genetic marker for improving resistance to the disease.

## 1. Introduction

Flystrike, or infestation of the fleece and skin with the larvae of various fly species, is a problem affecting the sheep industry globally, and the topic has been comprehensively reviewed recently [1]. It causes economic losses due to its negative impact on animal welfare and production performance, and there is increased cost associated with treating and preventing the disease. The occurrence of flystrike is environmentally dependent and its incidence and prevalence are variable, but there are several factors that make individual sheep more predisposed to flystrike, such as variation in the fleece, skin and immune response [2].

It has been determined that the immune system of a sheep can recognise the foreign components of a fly larva and produce an immune response to it [3], hence immune responses may play a role in defining flystrike resistance and susceptibility. However, using the Trangie research flocks in Australia, which were bred for resistance or susceptibility to fleece rot and flystrike, O’Meara et al. [4] found that antibody titres did not differ between the resistant and the susceptible animals. This led them to suggest that the resistance of sheep to fleece rot and flystrike was likely to be founded in the innate immune response, and not the adaptive response.

The toll-like receptors (TLRs) are a family of proteins that play a role in the innate immune response. Each TLR recognises particular patterns of pathogen-associated molecules, which can subsequently provoke innate immune responses [5]. The TLRs are widely expressed in immunologically active cells, including dendritic cells, macrophages, lymphocytes, and endothelial cells [6]. They are also classified into two subfamilies based on their localization on or in the cell: specifically the cell-surface TLRs and the intracellular TLRs [7].

The receptor TLR9 is an intracellular TLR and recognizes DNA containing unmethylated cytosine-phosphate-guanine (CpG) motifs [8]. Unmethylated CpG motifs are more prevalent in bacterial genomes than in vertebrate genomes, and their recognition by TLR9 triggers the activation of the mitogen-activated protein kinase pathway, nuclear factor kappa B, and multiple transcription and translation factors [9]. However, a recent study on 41 insect species from six orders revealed that DNA methylation was found in all orders investigated with the exception of the Diptera (flies), including *Lucilia cuprina*, one of the main species associated with flystrike in sheep [10].

Variation in human *TLR9* gene (*TRL9*) has been reported to be associated with variation in susceptibility to a diverse range of diseases, such as Crohn’s disease [11], lupus erythematosus [12], malaria [13] and talaromycosis [14]. In cattle, variation in *TLR9* has been associated with susceptibility to bovine tuberculosis [15], and in sheep it has been reported to be associated with seropositivity for small ruminant lentivirus in the Boutsko, Friesarta and Comisana breeds in Greece [16], and in 91 flocks of sheep reared in Italy [17]. Collectively these studies suggest a role for *TLR9* in the immune response to infectious and other diseases.

In this study, we collected blood samples from sheep with flystrike, and phenotypically normal sheep from the same flocks for a variety of sheep breeds farmed in New Zealand (NZ). We used polymerase chain reaction-single strand conformation polymorphism (PCR-SSCP; see [18] for a review of its applicability) to analyse variation in *TLR9* and looked for associations between variation in *TLR9* and the occurrence of flystrike.

## 2. Materials and Methods

### 2.1. Animals

One hundred and seventy-eight sheep that had flystrike or were recovering from flystrike were sampled from three strike seasons (2015–2017), and 134 sheep that had no symptoms of flystrike were also sampled from the same farms at the same time. These sheep were from both commercial and stud breeding operations throughout NZ and they were of different breeds, including: Merino, Corriedale, Perendale, Romney, Lincoln, Coopworth, Poll Dorset, Texel, Finn, Dorset Down, Suffolk, South Suffolk, Shropshire, and various crossbred sheep and composites. The sheep were from farms where the farmers had used a variety of management practices to control flystrike.

### 2.2. Identification of Flystrike

Sheep with flystrike, and those that had had flystrike, were identified either during shearing, or in the yard. These sheep were diagnosed as having active flystrike when larvae could be seen on the skin, and their wool was discoloured and bad smelling. Sheep that had had flystrike were identified through having areas of pink skin with little or no wool, obvious scar tissue from larval damage and/or flaky, dry skin.

### 2.3. Blood Collection

Blood samples from individual sheep were taken via ear nicking and collection onto FTA^TM^ cards (Whatman, Middlesex, UK). This approach to blood collection complies with the Animal Identification Section 7.5 of the Animal Welfare (Sheep and Beef Cattle) Code of Welfare 2010, which is a welfare issued code under the New Zealand Animal Welfare Act 1999 (New Zealand Government). In contemporary NZ farming, both blood and ear tissue samples are regularly collected for the purpose of animal breeding, and this can include the use of commercially available single-gene diagnostic tests for both disease and performance traits, and the use of genome wide screening approaches including SNP chip typing and whole genome sequencing. Accordingly, ethics approval was not directly required for this research.

Genomic DNA for the PCR amplifications was purified from the dried blood spots using a two-step procedure described by Zhou et al. [19].

### 2.4. PCR-SSCP Analysis and Genotyping of Ovine TLR9

A 414-bp fragment of ovine *TLR9* exon 2 that encodes for the Z-loop [20] and a putative CpG-DNA binding domain [21], was amplified using the PCR primers described in Zhou et al. [22]. These primers were 5′-ttcgtggacctgtcggac-3′ and 5′-ctggctgttgtagctgag-3′, and they were synthesised by Integrated DNA Technologies (Coralville, IA, USA).

PCR amplification was performed in a 15-μL reaction. This contained the purified genomic DNA on a 1.2 mm punch of the FTA^TM^ card, 0.25 μM of each primer, 150 μM dNTP’s (Bioline, London, UK), 2.5 mM of Mg^2+^, 0.5 U of Taq DNA polymerase (Qiagen, Hilden, Germany) and 1× the reaction buffer supplied with the enzyme. The thermal profile for amplification consisted of 2 min denaturation at 94 °C, followed by 34 cycles of 30 s at 94 °C, 30 s at 61 °C and 30 s at 72 °C, with a final extension of 5 min at 72 °C. Amplification was carried out in S1000 thermal cyclers (Bio-Rad, Hercules, CA, USA).

A 0.7-μL aliquot of each amplicon was mixed with 7 μL of loading dye (98% formamide, 10 mM EDTA, 0.025% bromophenol blue, 0.025% xylene-cyanol). After denaturation of the amplicon at 95 °C for 5 min, the samples were rapidly cooled on wet ice and then loaded on 16 cm × 18 cm, 14% acrylamide: bisacrylamide (37.5:1) (Bio-Rad) gels. Electrophoresis was performed using Protean II xi cells (Bio-Rad), at 300 V for 19 h at 23 °C in 0.5×TBE buffer. Polymerase chain reaction amplicons of three previously known alleles (*01, *02 and *03) of ovine *TLR9* [22] were run as standards to identify the *TLR9* alleles present in the individual samples. The gels were silver-stained according to the method of Byun et al. [23].

### 2.5. Sequencing of New Alleles and Sequence Analysis

Alleles that exhibited PCR-SSCP patterns different to those of the previous identified alleles *01, *02 and *03 were regarded as new alleles and were sequenced at the Lincoln University DNA Sequencing Facility. For those alleles that were only found in heterozygous samples, they were sequenced using an approach described previously [24]. Briefly, single bands of interest from the heterozygous were recovered directly from the SSCP gels as a gel slice. These were macerated and the DNA was eluted into 50 µL TE buffer by incubating at 70 °C for 20 min. One µL of the eluted solution was used as a template for PCR amplification to produce PCR amplicons with SSCP patterns that represented a hemizygous portion of the patterns derived from the original heterogeneous amplicons. These ‘homozygous’ PCR amplicons were then directly sequenced. Sequence alignment and comparisons were carried out using DNAMAN (version 5.2.10, Lynnon BioSoft, Vaudreuil, QC, Canada).

### 2.6. Statistical Analyses

All statistical analyses were performed using R Statistics version 3.6.0. First, univariate Pearson Chi-square tests were undertaken to explore the relationships between the known variables (age, gender, year, wool type, breed, farm, and region) and the occurrence of flystrike to ascertain whether any confounding factors needed to be adjusted for. Next, for each of the four *TRL9* alleles, a Pearson Chi-square test along with a binary logistic regression was performed to explore the relationship between the presence of each allele and the occurrence of flystrike, with a correction for the variables applied, if they were associated with the occurrence of the disease.

## 3. Results

### 3.1. Alleles of TLR9 and Their Frequencies 

**Figure 1:**
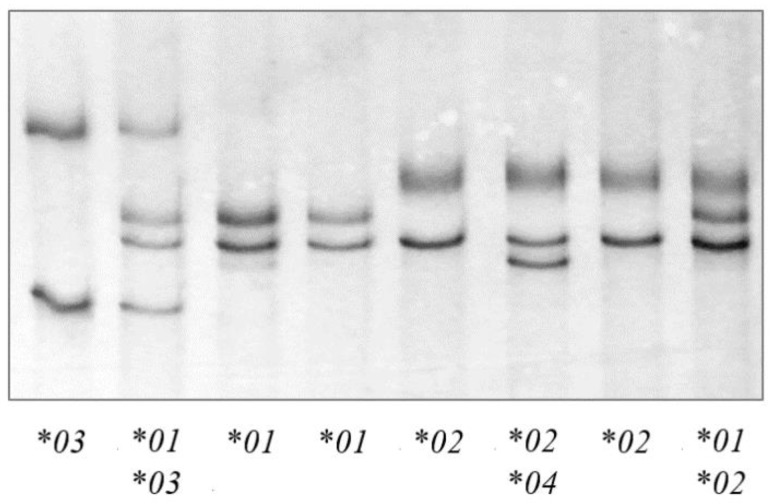
Polymerase chain reaction—single strand conformation polymorphism (PCR-SSCP) analysis of the ovine *TLR9* exon 2 region. Four unique banding patterns representing four alleles (*01, *02, *03 and *04) were observed in either homozygous or heterozygous forms.

**Figure 2:**
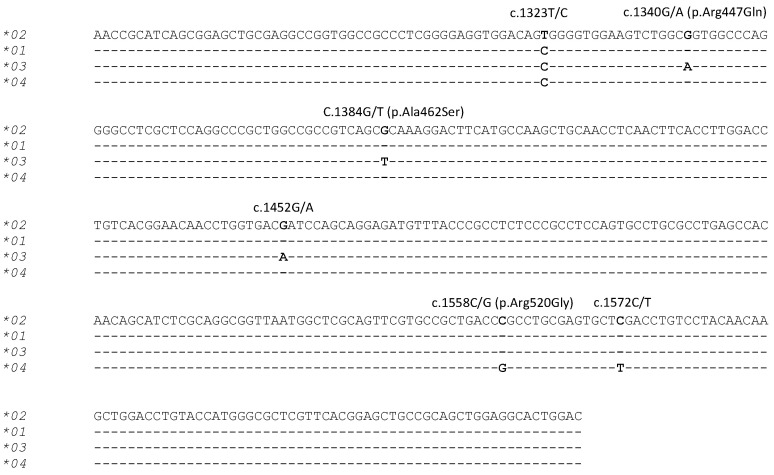
Alignment of the four ovine *TLR9* allele nucleotide sequences. Six SNPs identified in the three previously identified alleles (*01, *02 and *03) [22] and the newly identified allele (*04) are marked above the sequences with the SNPs marked in bold. The annotation of the SNPs and amino acid substitutions follows the HGVS nomenclature (http://www.hgvs.org/mutnomen/). The sequences exclude the PCR primer binding regions and dashes represent nucleotide sequences identical to the reference sequence (Allele *02).

PCR-SSCP analysis of the 312 sheep investigated revealed four unique banding patterns (*01 to *04) (Figure 1). The first three banding patterns matched those of the three previously identified alleles *01 to *03 [22], whereas the fourth patterns represented a new allele *04 (Figure 2). The identification of this new allele led to the addition of two further single nucleotide polymorphisms (SNPs) to the gene region investigated, including one synonymous SNP (c.1572C/T) and one non-synonymous SNP (c.1558C/G), which would led to the amino acid change p.Arg520Gly.

The number and percentage of sheep carrying the *TLR9* alleles in the flystrike and non-flystrike groups are shown in Table 1. Among the three common alleles, the largest difference between these two groups was observed for the percentage of sheep carrying allele *03. In the flystrike group, 17.4% of sheep carried *03, compared to 26.1% of sheep who carried *03 in the non-flystrike group.

### 3.2. Associations between the Variables and Flystrike Occurrence

The categories of the variables (age, gender, year, wool type, breed, farm, and region) and their frequencies are summarized in Supplementary Table S1. The univariate Pearson Chi-square tests between the variables (age, gender, year, wool type, breed, farm, and region) and the occurrence of flystrike are shown in Supplementary Table S2. No significant association was detected between these variables and flystrike occurrence, suggesting there was no need to correct for these variables.

Pearson Chi-square tests along with binary logistic regression using the occurrence of flystrike as dependent variable and the allele as independent variable, revealed a relationship between the presence of *TLR9* allele *03 and flystrike (Table 2), with the presence of allele *03 being associated with a lower likelihood of flystrike occurrence (OR = 0.499, *p* = 0.024).

## 4. Discussion

This study was undertaken to investigate whether variation in ovine *TLR9* was related to variation in flystrike susceptibility. A new allele sequence of *TLR9* was identified, which may be a consequence of more sheep from different sheep breeds being investigated in this study, when compared to the work of Zhou et al. [22].

The detection of this new allele brings the number of SNPs identified in this 378-bp fragment of exon 2 of *TLR9* (excluding the primer binding regions) from four to six, giving an average SNP density of 15.9 SNPs per kb of genome. This SNP density is comparable to that reported for other highly polymorphic genes, such as the keratin-associated protein genes *KRTAP28-1* [25] and *KRTAP36-1* [26]. It is notably higher than the average density of 4.9 SNPs per kb across the sheep genome suggested by Kijas et al. [27], such that delineating this variation using tools like the Illumina OvineSNP50 DNA Analysis Kits (Illumina Inc., San Diego CA, USA) may be difficult. Even with the claimed 54,241 evenly spaced probes in this kit, and the assertion that this is sufficient SNP density for genome-wide association studies, it is still only detecting an average one SNP per 55 kb, compared to the one SNP per 63 bp in the four different coding region sequences described here. In this respect it is notable that half of the SNPs identified in *TLR9* are non-synonymous, and that this may be important for the function of the receptor.

Of the three common alleles in the sheep studied, association with occurrence of flystrike was only detected for allele *03. This allele contained three unique nucleotide sequences at positions c.1340, c.1384 and c.1452, when compared to alleles *01 and *02. This suggests that the SNPs at these positions may have effect on the function of the receptor. Unlike many other TLRs, TLR9 possesses a loop region (known as the Z-loop) composed of approximately 38 amino acid residues [20]. Proteolytic processing at the Z-loop has been revealed to be necessary for creation of a functional TLR9 protein [28]. In this respect, the SNPs c.1340 and c.1384 are located within the Z-loop encoding region of the gene, and if translated, would lead to the amino acid changes p.Arg446Gln and p.Ala462Ser. These amino acid changes may affect the structure or proteolytic cleavage of TLR9, and consequently have functional effect on its binding activity and function in immune responses.

Additionally, the potential effect of the synonymous SNP at c.1452 cannot be ignored, as synonymous SNPs have been reported to influence mRNA stability and translation [29], and/or protein folding [30,31], or affect gene expression via codon preference differences, thereby potentially resulting in altered expression levels [17,30].

Alternatively, the SNPs detected here may not have any functional effect by themselves, but may be in linkage with variation in other regions of the gene, or nearby genes with functional or structural significance. In this respect, in a study of the Trangie sheep, Smith et al. [2] suggested that the genes for fibulin (*FBLN1*) and fatty-acid binding protein 4 (*FABP4*) are associated with to resistance to fleece rot in Australian Merino sheep. Subsequently, Burrows et al. [32] typed ovine *FABP4* and confirmed an association between the gene and flystrike susceptibility.

In the context of the association revealed here, *TLR9* (ENSOARG00000006162) is located on ovine chromosome 19, while *FABP4* (ENSOARG00000009344) is on chromosome 9 and *FBLN1* (ENSOARG00020001970) is on chromosome 3, suggesting that the association revealed with *TLR9* is unlikely to be because of the latter two genes. The *TLR9* gene is in proximity to the twinfilin actin binding protein 2 gene (*TWF2*: ENSOARG00000005975) on ovine chromosome 19, but this actin-binding protein is thought to be involved in motile and morphological processes in mammals, thus it having a role in susceptibility to flystrike seems unlikely.

## 5. Conclusions

Four alleles of *TLR9* were detected in 312 sheep genomes, of which *04 was found for the first time, and *02 was the dominant allele in the sheep studied, followed by *03. The presence of *TRL9* *03 was found to be associated with a lower likelihood of sheep having flystrike. If allele *03 is an effective molecular marker for selection of flystrike resistance in sheep, then it could potentially be used as a genetic marker for breeding, but to be confident of this approach the relationship between *TLR9* variation and susceptibility needs to be tested more widely.

## Figures and Tables

**Table 1:** Number and percentage of animals carrying individual *TLR9* alleles, for sheep with and without.

Allele	Sheep with Flystrike (*n* = 178)		Sheep without Flystrike (*n* = 134)	
	Number of Sheep Carrying the Allele	Percentage of Sheep Carrying the Allele (%)	Number of Sheep Carrying the Allele	Percentage of Sheep Carrying the Allele (%)
*01	28	15.7	14	10.4
*02	164	92.1	128	95.5
*03	31	17.4	35	26.1
*04	6	3.4	7	5.2

**Table 2:** The association of the presence of each *TRL9* allele with the occurrence of flystrike.

Allele	Odds Ratio ^1^	95% Confidence Interval		*p*-Value
		Lower	Upper	
*01	1.195	0.575	2.566	0.638
*02	0.414	0.126	1.241	0.126
*03	0.495	0.268	0.900	**0.022**
*04	0.585	0.182	1.824	0.351

^1^ Odds ratio = p/(1 − p) calculated by exp(coefficient) in a logistic regression model, where the dependent variable is the presence or absence of flystrike, and the independent variable is the presence or absence of the allele. *p* < 0.05 in bold.

## Data Availability

The data presented in this study are available on request from the corresponding authors.

## Supplementary Materials

The following are available online at https://www.mdpi.com/article/10.3390/ani11123549/s1, Table S1: Categorisation of the variables; Table S2: Pearson Chi-square analyses exploring associations between the variables and flystrike occurrence.
