# Supplementary material for: Ovine Toll-like Receptor 9 (TLR9) Gene Variation and Its Association with Flystrike Susceptibility
Source: Animals (Basel). 2021 Dec 14;11(12):3549. doi: 10.3390/ani11123549 (PMC8697942; doi:10.3390/ani11123549)
Supplement: Supplementary file 1 [file animals-11-03549-s001.zip › animals-1491759-supplementary.pdf]

animals

MDPI

Supplementary material

Ovine Toll-like Receptor 9 (*TLR9*) Gene Variation and Its Association with Flystrike Susceptibility

Xiu Liu [1], Huitong Zhou [2], Hua Gong [2], Wenting Liu [3], Qian Fang [2], Yuzhu Luo [1,*], Jiqing Wang [1], Shaobin Li [1], Jiang Hu [1] and Jonathan G.H. Hickford [2,*]

1 Gansu Key Laboratory of Herbivorous Animal Biotechnology, College of Animal Science and Technology, Gansu Agricultural University, Lanzhou 730070, China; liuxiu@gsau.edu.cn (X.L.); wangjq@gsau.edu.cn (J.W.); lisb@gsau.edu.cn (S.L.); huj@gsau.edu.cn (J.H.)
2 Department of Agricultural Sciences, Faculty of Agriculture and Life Sciences, Lincoln University, Lincoln 7647, New Zealand; Huitong.Zhou@lincoln.ac.nz (H.Z.); gonghua3000@gmail.com (H.G.); fangq@lincoln.ac.nz (Q.F.)
3 School of Public Health, Hubei University of Medicine, Shiyan 442000, China; liuwentingnz@outlook.com
* Correspondence: luoyz@gsau.edu.cn (Y.L.); Jonathan.hickford@lincoln.ac.nz (J.G.H.H.); Tel.: +86-931-763-1870 (Y.L.); +64-3423-0665 (J.G.H.H.)

Citation: Liu, X.; Zhou, H.; Gong, H.; Liu, W.; Fang, Q.; Luo, Y.; Wang, J.; Li, S.; Hu, J.; Hickford, J.G.H. Ovine Toll-like Receptor 9 (TLR9) Gene Variation and Its Association with Flystrike Susceptibility. *Animals* **2021**, *11*, 3549. https://doi.org/10.3390/ani11123549

Academic Editor: Esmaeil Ebrahimie and Maria Luisa Dettori

Received: 12 October 2021
Accepted: 3 December 2021
Published: 14 December 2021

Publisher's Note: MDPI stays neutral with regard to jurisdictional claims in published maps and institutional affiliations.

Copyright: © 2021 by the authors. Licensee MDPI, Basel, Switzerland. This article is an open access article distributed under the terms and conditions of the Creative Commons Attribution (CC BY) license (https://creativecommons.org/licenses/by/4.0/).

Table S1. Categorisation of the variables.

Variable	Categories	Frequencies
**Age**	1	102
	2	81
	3	41
	4	88
**Gender**	Ewe	160
	Ram	38
	Unknown	114
**Year**	2015	121
	2016	156
	2017	35
**Wool Type**	Down wool	53
	Fine wool	126
	Strong wool	133
**Breed**	Composite	6
	Coopworth	31
	Corriedale	61
	Crossbred	44
	Dorset Down	18
	Lincoln	7
	Merino	65
	Perendale	9
	Romney	36
	Shropshire	12
	South Down	1
	South Suffolk	6
	Suffolk	13
	Texel	3
**Farm**	Ashley Dene	19
	Bob Masefield	4
	Chris Hampton	16
	Dave Clark	4
	Doc Sidey	7
	Fliss Gardiner	3
	Helen Heddell	60
	Hugh Taylor	1
	Ike Williams	8
	Ike Williams	15
	Lincoln University	56
	Neville Moorhead	32
	Paul Gardiner	3
	Penni Loffhagen	8
**Region**	Banks Peninsula	4
	Mid Canterbury	124
	North Canterbury	16
	Northland	16
	South Canterbury	152

Table S2. Pearson Chi-square analyses exploring associations between the variables and flystrike occurrence.

Variable	Flystrike
Age	0.3441
Gender	0.3646
Year	0.7826
Wool type	0.8374
Breed	0.9118
Farm	0.9191
Region	0.3886
